# Mitigating Wetting and Scaling in Air Gap Membrane Distillation Crystallization via SiO_2_ Seeding

**DOI:** 10.3390/membranes15100321

**Published:** 2025-10-17

**Authors:** Stefanie Flatscher, Mark W. Hlawitschka, Wolfgang M. Samhaber, Florian Hell, Josef Lahnsteiner

**Affiliations:** 1 Institute of Process Engineering, Johannes Kepler University Linz, Altenberger Straße 69, 4040 Linz, Austria; 2 VA Tech Wabag GmbH, Dresdner Strasse 89-91, 1200 Vienna, Austria; florian.hell@wabag.com (F.H.); josef.Lahnsteiner@wabag.com (J.L.)

**Keywords:** air gap membrane distillation, seeding crystallization, membrane wetting

## Abstract

Membrane distillation crystallization (MDCr) is an approach for treating hypersaline wastewaters and enabling zero-liquid-discharge (ZLD) systems. However, its performance is often inhibited by concentration polarization, scaling, and membrane wetting. Heterogeneous seeding has been proposed to shift crystallization into the bulk phase, yet its quantitative influence on flux stability, wetting resistance, and crystal growth remains poorly understood. This study investigates air-gap MDCr (AGMDCr) of 300 g L^−1^ NaCl using polypropylene (PP) and polytetrafluoroethylene (PTFE) membranes under seeded and unseeded conditions. Introducing 0.1 g L^−1^ SiO_2_ seeds (30–60 µm) enhanced steady-state permeate flux by 41% and maintained salt rejection ≥ 99.99%, indicating effective suppression of wetting. Seeding shifted the crystal size distribution from fine (mean 50.6 µm, unseeded) to coarse (230–340 µm), consistent with reduced primary nucleation and preferential growth on seed surfaces. At 0.6 g L^−1^, the flux decreased relative to 0.1–0.3 g L^−1^, consistent with near-wall solids holdup and hindered transport at high seeding concentration. The PTFE membrane exhibited a 47% higher flux than PP, primarily due to its reduced thermal resistance and optimized module geometry at the same flow rate. These results demonstrate that appropriately sized and dosed SiO_2_ seeding effectively stabilizes flux and suppresses wetting in MDCr.

## 1. Introduction

Freshwater scarcity is a rising global challenge, driven by population growth, industrialization, and climate change. According to the United Nations, over two billion people already live in regions under high water stress, a figure projected to increase in the coming decades [1,2]. Conventional desalination technologies, such as reverse osmosis, have expanded water supply but generate large volumes of concentrated brine. Discharge of this hypersaline effluent can disrupt marine ecosystems, alter local hydrodynamics, and degrade aquatic biodiversity [3,4,5,6]. As climate change and water scarcity intensify, the environmental burden of brine disposal is pushing the development of advanced water treatment technologies that enable both freshwater production and sustainable brine management. Among these, thermally driven processes such as membrane distillation (MD) offer a promising alternative, providing high salt rejection and tolerance to hypersaline feeds [7]. Unlike pressure-driven processes such as reverse osmosis, where the required hydraulic pressure becomes prohibitively high for hypersaline brines due to osmotic limits, MD’s driving force is a temperature-induced vapor pressure gradient, largely unaffected by feed salinity. This makes it uniquely suited for treating brines near saturation using low-grade or renewable heat sources (40–80 °C) [8,9,10,11]. When integrated with crystallization (MDCr), these systems not only recover high-purity water but also enable controlled precipitation of dissolved salts, contributing to zero-liquid-discharge (ZLD) strategies and resource recovery from industrial and municipal brines [12,13,14,15,16,17]. However, MDCr performance is limited by severe concentration and temperature polarization (CP, TP), membrane scaling, and wetting [16,18,19]. CP reduces the effective driving force for vapor transport, while TP alters the temperature at the membrane interface, lowering transmembrane vapor pressure. Scaling, particularly under hypersaline conditions, forms crystalline deposits on the membrane surface that block vapor pathways and promote wetting. Various hydrodynamic and interfacial strategies have been proposed to mitigate these effects, including spacers [20], two-phase flow [21,22], and the addition of inert particles [23]. These insoluble seeding particles serve as preferential nucleation sites, promoting crystallization away from the membrane surface, and thereby mitigating scale formation and wetting [18,23,24,25,26].

The quantitative influence of seeding parameters on air gap MDCr (AGMDCr) performance remains poorly resolved. Most existing studies have investigated seeding under a narrow set of operating conditions, without systematically evaluating how seed concentration affects flux stability, wetting thresholds, and long-term performance under hypersaline operation [25,27]. The role of seed particle size in modulating crystal size distribution (CSD) within the continuous supersaturation environment of MDCr, where particle, membrane, and particle–particle interactions are complex, has not been clearly established [18,28]. Furthermore, the interplay between seed properties and hydrodynamic regime, particularly in air gap configurations, has received little attention, despite its direct impact on polarization phenomena and vapor transport efficiency.

Comparative analyses across membrane types are rare. Polypropylene (PP) and polytetrafluoroethylene (PTFE) are the two most common commercial MD membranes. Their response to seeding, especially under AGMDCr conditions, remains largely undocumented. This limits the ability to generalize scaling-control strategies and optimize MDCr for ZLD applications across different material systems [14,24,26,29].

Despite these advances, critical knowledge gaps persist that hinder the optimization and scale-up of seeded AGMDCr. A quantitative and systematic understanding of how seeding parameters like concentration and seed size influence flux stability and wetting thresholds is notably absent. This issue is intrinsically linked to an inadequate understanding of the interplay between these seed particles and the hydrodynamic regime, particularly the resulting impact on polarization and vapor transport.

To address these gaps, this study provides the first systematic investigation into the effects of SiO_2_ seeding parameters (concentration and size) and hydrodynamics on AGMDCr performance. Uniquely, a direct comparative analysis using both commercial PP and PTFE membranes was conducted under identical hypersaline conditions. The aim of this work is therefore not only to quantify the influence of seeding but also to establish a mechanistic framework that links seed properties, hydrodynamics, and membrane material to flux stability and wetting resistance. The results provide a crucial foundation for designing robust, scalable MDCr processes for ZLD and resource recovery.

## 2. Materials and Methods

### 2.1. Experimental Setup

**Figure 1:**
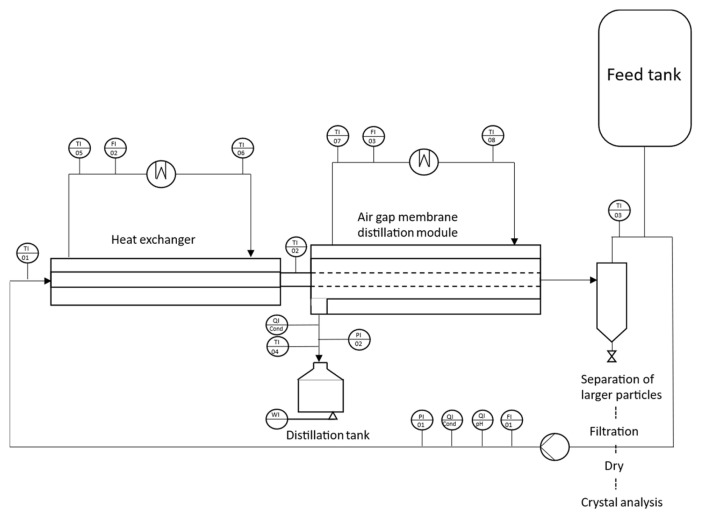
Schematic sketch of a mini-AGMDCr plant with a heat exchanger module, a membrane module, and a classifier part.

All experiments were conducted using a mini-pilot-scale AGMDCr system (Figure 1). The setup comprised a tubular membrane module, a tubular heat exchanger with a graphite-reinforced PP tube (outer diameter 10 mm, wall thickness 1 mm, Technoform, Kassel, Germany), and a vertical sedimentation tube. The membrane and heat exchanger modules were positioned horizontally in all experiments.

The feed solution was preheated in a counter-current heat exchanger (Haake, NB 22, Karlsruhe, Germany), thermostatically controlled to the target temperature, and pumped into the inner diameter of the tubular membrane. The feed flow was supplied by an air-operated diaphragm pump (VerderAIR, VA08, Vleuten, Netherlands), with the volumetric flow rate monitored by a magnetic flow meter (Kobold, MIK 5N15AL343, Hofheim am Taunus, Germany). The cold-side stream flowed counter-currently through the condensation channel, with the condensed permeate collected in a 5 L glass tank and continuously weighed on a balance (Kern, DE60K2N, Balingen, Germany). Resistance thermometers (Kobold, MWE PT100) recorded the inlet and outlet temperatures of the membrane module, the temperature of the permeate in the collection vessel, and the inlet/outlet temperatures of both hot and cold heat exchangers. Conductivity was continuously monitored in the concentrating loop using a conductivity meter (Kobold, LCI SG40MPF). At the same time, pH was measured with a gel-filled, in situ pH electrode, and the permeate was analyzed using an inline conductivity meter (Kobold, ACS-Z2T1G).

The membrane module’s cold side consisted of a copper condensation tube (outer diameter 20 mm, wall thickness 1 mm) separated from the membrane by a 4 mm air gap. Tap water (20 ± 1.5 °C) was used as the coolant. The system was operated in batch mode, with the feed solution recirculating continuously until the end of the experiment. All process parameters and permeate mass were recorded every minute.

The feed solution for each experiment was prepared with 300 g L^−1^ (23.08 wt%) NaCl and 1 L of deionized water. The feed inlet temperature was 53 ± 0.5 °C, and the cold side temperature was 20 ± 1.5 °C. The circulating feed flow was 95 ± 5 L h^−1^, corresponding to linear velocities of 0.56 m s^−1^ (PTFE module) and 1.167 m s^−1^ (PP module). Each run was performed in batch mode and stopped after 6 h.

Two commercially available hydrophobic tubular membranes were evaluated in AGMDCr configuration: a PP membrane (3M^®^, Accurel PP V8/2 HF, Neuss, Germany) and an expanded polytetrafluoroethylene (ePTFE) membrane (Teflex Gasket, Cixi City, China). Key geometric and physical properties are summarized in Table 1.

The PTFE membrane’s larger inner diameter, reduced wall thickness, and comparable or higher porosity provide a lower transport resistance and higher vapor-throughput capacity than the PP module under identical conditions.

### 2.2. Seeding Materials and Experimental Procedure

Quartz sand (SiO_2_, purity > 99%) was used as an inert heterogeneous nucleant for this study. This material was chosen for its combination of low cost, global availability, and high physicochemical stability. It is chemically stable and insoluble in aqueous solutions, ensuring that it acts purely as a preferential nucleation site without altering the brine chemistry, a distinct advantage over soluble seeds. Three size fractions were investigated: 30–60 µm, 75–125 µm, and 210–300 µm. Seeds were dispersed directly into the feed vessel before startup and were maintained in suspension by recirculation. Larger crystals formed during operation were removed in the sedimentation tube. Four sets of experiments were performed:

1. Optimizing AGMDCr process (PP and PTFE): A comparative analysis was performed between the unseeded process and a process seeded with a fixed SiO_2_ concentration of 0.1 g L^−1^. Key performance indicators, including permeate flux stability, membrane wetting and scaling behavior, and overall water recovery, were evaluated to determine the effect of seeding.
2. Seeding concentration series (PP and PTFE): SiO_2_ concentrations of 0, 0.1, 0.3, and 0.6 g L^−1^ were tested to assess effects on permeate flux stability, wetting behavior, and product crystal size distribution (CSD).
3. Seed-size series (PTFE only): At a fixed SiO_2_ concentration of 0.1 g L^−1^, the effect of seed particle size was evaluated using three distinct fractions: 30–60 µm, 75–125 µm, and 210–300 µm. The analysis focused on the resulting changes in the CSD.
4. Hydrodynamic series (PTFE only): At fixed SiO_2_ concentration of 0.1 g L^−1^ (30–60 µm), Reynolds numbers (Re) of 3366, 3927, and 4488 (transition to turbulent internal flow) were imposed by adjusting the feed flow rate $\dot{Q}$, while keeping the other parameters constant. For circular-tube flow, the Re number was estimated by
  (1) $$Re=\frac{\;\;\rho \;u\;D_{h}}{\mu },$$
  and
  (2) $$u=\frac{4\;\dot{Q}}{\pi D^{2}},$$
  where u is the bulk velocity, D the inner diameter (for a circular tube, the hydraulic diameter $D_{h}$ = D), and ρ and μ are the density and dynamic viscosity of the 300 g L^−1^ NaCl solution evaluated at the bulk feed temperature (53 ± 0.5 °C). Setpoints were reached by tuning $\dot{Q}$ and verified from the recorded flow.

Hydrodynamic experiments were conducted only with the PTFE module at a fixed seeding condition of SiO_2_ 0.1 g L^−1^ (30–60 µm), added before start-up. All other operating parameters were identical to Section 2.1. All conditions were performed in triplicate (*n* = 3).

### 2.3. Performance Evaluation

Permeate flux was obtained gravimetrically and reported as a mass flux with

(3) $$J=\frac{∆m}{A\;\cdot ∆t},$$

where J is the permeate flux, ∆m is the increase in permeate mass over the interval ∆t and A is the effective membrane area taken from Table 1.

Salt rejection quantified selectivity is derived by

(4) $$R=\left(1-\frac{C_{p}}{C_{f}}\right)\;\;\cdot \;100\%,$$

where $C_{f}$ is the feed concentration and $C_{p}$ the permeate concentration.

Water recovery was computed on a mass basis from the gravimetrically recorded permeate mass $M_{p}(t)$ and the initial feed mass $M_{f,0}$

(5) $$WR\left(t\right)=\frac{M_{p}\left(t\right)}{M_{f,0}}\;\cdot 100\%,$$

$M_{f,0}$ was obtained from the initial fill.

A run was classified as wetting-free if R is ≥99.99% and no rapid increase in permeate conductivity occurred. Partial wetting was flagged a priori if permeate conductivity rose from ≤10 µS cm^−1^ to ≥40 µS cm^−1^.

Scaling was diagnosed when a significant flux decline (≥10–20% within ≤60 min) occurred without a connected rise in permeate conductivity, and was verified, where applicable, by a persistent loss of flow at constant pump setting.

### 2.4. Crystal Sampling and Analysis

Crystal formation was monitored in situ in the sedimentation tube using an imaging setup consisting of an Allied Vision Allvium GigE camera and an Edmund Optics telecentric objective lens (1.0 × magnification, 110 mm, 63,731). Crystal size distributions (CSDs) were quantified from these 2D images using ImageJ (v1.54h). Equivalent-circle diameters were computed on a sample basis, with >1000 individual crystals analyzed. To complement size distribution analysis, selected dried crystal samples (filtered with Whatman 593 and dried at ambient conditions) were imaged using a digital microscope (Keyence, VHX-7000) for qualitative assessment of crystal morphology and habit.

No crystal sampling or imaging was performed for the hydrodynamic experiments. This series was used exclusively to quantify flux, salt rejection, wetting, and scaling based on process signals.

### 2.5. Post-Run Membrane Surface Morphology

To evaluate interfacial scaling at the membrane–liquid interface, PTFE membrane parts were examined by digital microscopy (Keyence, VHX-7000). Pristine membrane and membrane retrieved after AGMDCr operation under 0.1 g L^−1^ SiO_2_ seeding and unseeded conditions were imaged. For each condition, multiple non-overlapping fields of view were recorded to enable qualitative comparison of deposit coverage, surface features, and defects. Imaging was conducted without additional sample preparation. Only PTFE membranes were analyzed by the microscope, as the PP membrane samples were no longer available.

## 3. Results and Discussion

### 3.1. Optimizing AGMDCr Process

**Figure 2:**
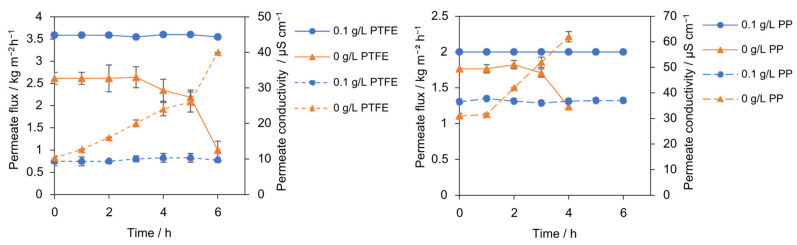
Permeate flux (solid lines) and permeate conductivity (dotted lines) with PTFE and PP membrane for seeded (0.1 g L^−1^, 30–60 µm, blue) and unseeded (orange) conditions.

The influence of minimal heterogeneous seeding particles on PTFE and PP membrane stability over time was assessed in 6 h runs with 300 g L^−1^ NaCl conducted unseeded and with 0.1 g L^−1^ SiO_2_ (30–60 µm) under identical conditions. The comparison of seeded and unseeded runs indicates that a minimal heterogeneous seeding (SiO_2_, 0.1 g L^−1^, 30–60 µm) sustains a high, time-stable vapor flux and preserves salt rejection over 6 h. In contrast, the unseeded control exhibits progressive flux decline (Figure 2). In the unseeded case, J(t) remains steady initially and then declines after 3 h from 2.64 to 1 kg m^−2^ h^−1^ by 6 h (−62%). During this time, permeate conductivity increased from 12 to 40 µS cm^−1^ for the PTFE membrane, reaching the 40 µS cm^−1^ wetting threshold after 6 h. Similarly, the PP membrane showed a flux decline from 1.82 to 1.23 kg m^−2^ h^−1^ (−32%), with its conductivity increasing from 31.5 to 62 µS cm^−1^ after 2 h. This indicates progressive wetting, or gradual pore intrusion, rather than an abrupt event. The sequencing in the unseeded run, conductivity rising well before the major flux decline, implicates progressive wetting as the dominant failure mode under these conditions. The plausible mechanism is that, in the absence of inert nuclei, interfacial supersaturation at the membrane remains elevated, favoring nucleation at or within the pores. Once intrusion initiates, the effective vapor-pressure driving force collapses locally, and the flux decays. In stark contrast, the addition of 0.1 g L^−1^ SiO_2_ stabilized the performance of both membranes, demonstrating wetting-free operation. The PTFE membrane maintained a high, stable flux of 3.51 kg m^−2^ h^−1^ with a low conductivity of 9 µS cm^−1^. The PP membrane also exhibited stable operation, maintaining a flux of 2 kg m^−2^ h^−1^ and a conductivity of 36 µS cm^−1^. SiO_2_ provides a distributed growth surface in the bulk and possibly increases near-wall mixing. Shifting the crystallization away from the membrane interface, limiting interfacial resistance, and suppressing pore intrusion [24].

**Figure 3:**
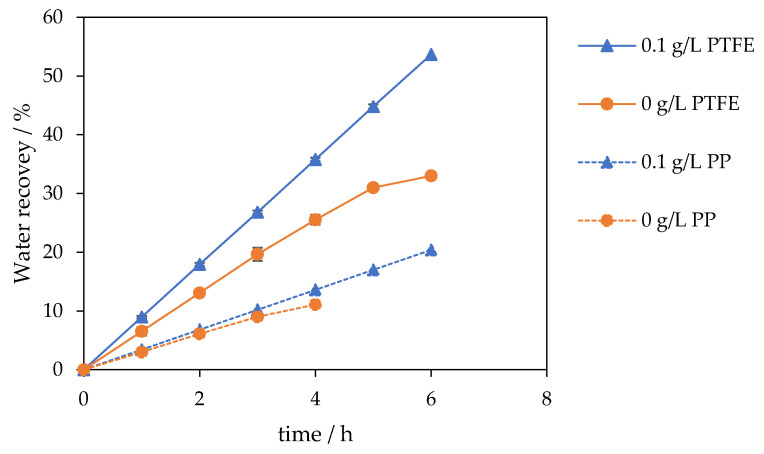
Water recovery for seeded (0.1 g L^−1^, 30–60 µm, blue triangles) and unseeded experiment (orange circles). Seeding increases water recovery: PTFE 54% vs. 33% (solid line) and PP 20% vs. 11% (dotted line) after 6 h.

The result is a higher, time-stable flux and a steeper, sustained water recovery slope over 6 h, achieved without compromising permeate quality. Water recovery curves mirrored these trends: the unseeded case rose linearly to approximately 20% and then plateaued at 33% by 6 h, whereas seeding maintained a steeper, near-linear increase to 54% at 6 h. A similar improvement was observed for the PP membrane; the unseeded experiment reached only 11% recovery after 4 h, while the seeded experiment achieved a stable 20% recovery after 6 h (Figure 3).

### 3.2. Seeding Concentration Series and Crystal Size Distribution (CSD)

**Figure 4:**
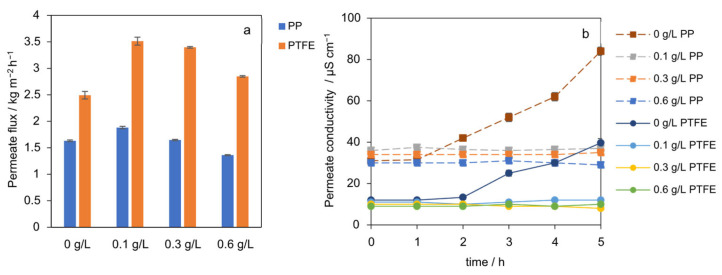
(**a**) Time-average mass flux J vs. SiO_2_ concentration (0 to 0.6 g L^−1^) for PTFE and PP membranes. Flux peaks at 0.1 g L^−1^, higher concentrations reduce performance. (**b**) The permeate conductivity over time for different seeding concentrations for PP and PTFE membranes.

The effect of SiO_2_ seed concentration on the permeate flux for both PP and PTFE membranes is shown in Figure 4a. The results reveal a non-linear relationship, indicating a local optimum seeding concentration around 0.1 g L^−1^ for maximizing performance in the AGMDCr system. The permeate flux in the absence of seeding particles is the lowest at 2.5 kg m^−2^ h^−1^ and 1.63 kg m^−2^ h^−1^, respectively. With 0.1 g L^−1^ seeding, the flux significantly increases to 3.51 kg m^−2^ h^−1^ (PTFE) and 1.92 kg m^−2^ h^−1^ (PP). However, increasing the seed concentration further to 0.6 g L^−1^ was disadvantageous. A reduction of 17% (PTFE) and 27% (PP) in the permeate flux was observed. At identical thermal conditions, adding 0.1 g L^−1^ SiO_2_ increased the time-average flux from 2.5 to 3.51 kg m^−2^ h^−1^ and from 1.63 to 1.92 kg m^−2^ h^−1^ on PP. As shown in Figure 4b, permeate conductivity remained low and stable for all seeded concentrations, confirming that wetting was not the cause of the observed flux changes. This contrasts with the unseeded case, where conductivity began to increase after 2 h, indicative of wetting. The improvement at low concentration is consistent with particle-induced thinning and mixing of the near-wall concentration layer, as well as preferential nucleation and growth in the bulk, which keeps the membrane interface below the spontaneous nucleation limit and reduces interfacial resistance. This effect is consistent with increased particle holdup near the membrane, which forms transient particulate layers that screen the surface and hinder vapor transport, as has been observed in other membrane systems [24,25].

Experiments with the PP membrane show the same qualitative trend as for PTFE but lower flux across all seeding concentrations (−41% at 0.1 g L^−1^). This gap is best explained by the membrane geometry, a smaller hydraulic diameter, and a thicker membrane wall, raising the overall heat-transfer and polarization resistances, rather than by an intrinsic polymer effect under these conditions. Taken together, the data indicate a local optimum at 0.1 g L^−1^ for this configuration while preserving wetting resistance. Higher loadings negate the polarization benefit and do not improve performance.

Mechanistically, the improvement at low seeding concentrations is consistent with particle-induced mixing of the boundary layers that weakens concentration polarization and shifts nucleation away from the membrane surface [23], whereas the decline at high seeding concentrations is consistent with emergent particulate layers that obstruct vapor transport.

**Figure 5:**
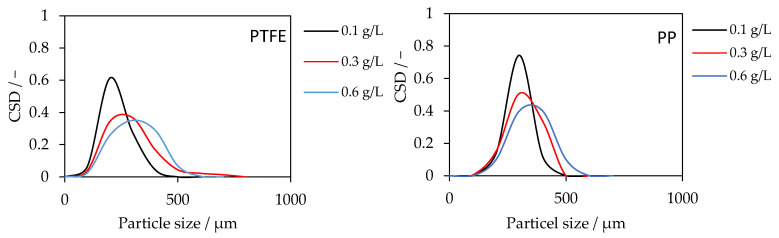
Crystal size distributions for PTFE (**left**) and PP (**right**) membranes at SiO_2_ seeding concentrations of 0.1, 0.3, and 0.6 g L^−1^. Increasing seeding concentration shifted the CSD to larger sizes. PP yields larger crystals than PTFE under identical conditions.

CSDs were obtained at the end of each run for PTFE and PP membranes with SiO_2_ = 0.1, 0.3, and 0.6 g L^−1^ (Figure 5). For PTFE, increasing the SiO_2_ loading shifts the CSD to larger sizes and broadens the distribution. At 0.1 g L^−1^, 80% of the particles lie within 114–231 µm (mean *d* = 172 µm). At 0.3 g L^−1^, the 80% interval shifts to 150–313 µm (*d* = 231 µm). At 0.6 g L^−1^, the distribution widens further to 176–347 µm (*d* = 261 µm), indicating a larger tail toward coarse size. For PP, the same monotonic shift is observed, and absolute sizes are higher at each concentration. At 0.1 g L^−1^, 80% fall in 193–291 µm (*d* = 242 µm); at 0.3 g L^−1^, 209–327 µm (*d* = 268 µm); and at 0.6 g L^−1^ in 225–375 µm (*d* = 300 µm).

The rightward shift with increasing seed concentration is consistent with earlier and more numerous heterogeneous nucleation events on seed surfaces, which extend the effective growth time of product crystals before sampling and suppress late secondary nucleation [28]. The broadening at 0.6 g L^−1^ suggests the onset of collision-mediated agglomeration/bridging, producing coarser clusters alongside primary crystals, this aligns with the flux decline observed at 0.6 g L^−1^ (added particulate resistance near the membrane interface). The consistently larger crystal sizes in PP compared to PTFE likely arise from membrane-specific geometric and structural factors. The PP membrane has a smaller inner diameter and greater wall thickness, resulting in higher shear and differing flow residence time in the sedimentation tube. These hydrodynamic differences influence the collision frequency and suspension stability, thereby promoting the formation of larger agglomerates. This underlines that seeding effects depend not only on seed concentration but also on membrane geometry and transport conditions, an aspect warranting further study.

### 3.3. Seed-Size Series and CSD

**Figure 6:**
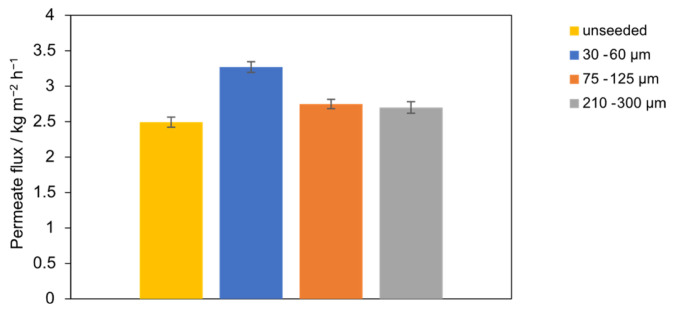
Permeate flux with the PTFE membrane for different seeding particle sizes (30–60 µm, 75–125 µm, and 210–300 µm) compared to unseeded operation.

This subsection evaluates the effect of seed sizes at a fixed SiO_2_ concentration of 0.1 g L^−1^ in the PTFE module (fractions: 30–60, 75–125, 210–300 µm; unseeded baseline). Figure 6 shows the time-average mass flux *J*. The highest *J* is obtained with seeds of 30–60 µm. Flux decreases monotonically with increasing seed size, with the 210–300 µm case 17% lower than the 30–60 µm case. The unseeded baseline yields the lowest flux, 24% below the 30–60 µm case. Permeate conductivity remains constant at 9 ± 2 µS cm^−1^ across all seed sizes, and salt rejection is ≥99.99%, indicating wetting-free operation; thus, the flux differences arise from transport effects rather than pore wetting. At fixed mass concentration, reducing seed size increases particle number density and total seed surface area. The denser population of small seeds provides more heterogeneous growth sites in the bulk and more effectively perturbs the near-wall concentration boundary layer, consistent with lower concentration polarization and higher flux. Conversely, larger seeds provide less total surface area and weaker near-wall disturbance, permitting higher interfacial supersaturation and slightly greater boundary-layer resistance, which explains the observed flux decline without loss of rejection. These trends align with seeding fundamentals (surface-area control of nucleation/growth and PSD) and with MD studies where insoluble particles weaken polarization up to an optimum window [18,28,30].

**Figure 7:**
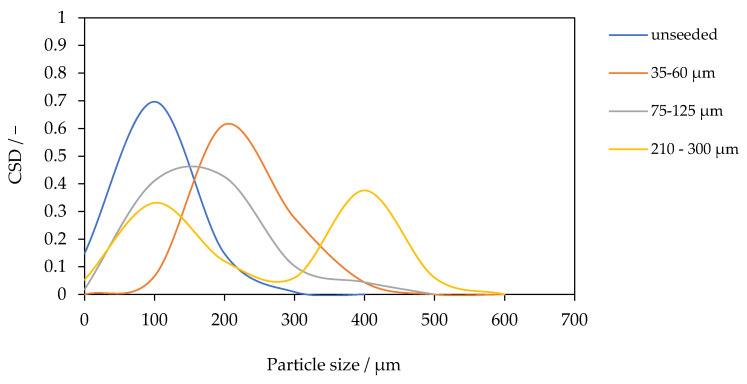
Crystal size distribution at a fixed SiO_2_ concentration of 0.1 g L^−1^ for different seed sizes (30–60 µm, 75–125 µm, and 210–300 µm) compared to unseeded operation.

The CSD for the PTFE module at 0.1 g L^−1^ SiO_2_ is shown in Figure 7 for seeds with diameters of 30–60 µm, 75–125 µm, and 210–300 µm, as well as for the unseeded baseline. The unseeded case is the finest: 80% of crystals lie between 10 and 94 µm with a mean of 50.6 µm. With 30–60 µm seeds, the distribution shifts strongly to coarser product (mean 172.2 µm). With 75–125 µm seeds, the mean is 116.9 µm, i.e., intermediate between unseeded and the smallest-seed case. For seeds with diameters of 210–300 µm, the CSD is bimodal, exhibiting a primary mode at approximately 71 µm and a secondary coarse mode at approximately 400 µm.

These results highlight that seed size exerts a non-monotonic influence on CSD. At fixed mass loading, smaller seeds (30–60 µm) provide the largest total surface area and highest number density of growth sites, leading to rapid consumption of supersaturation and growth-dominated crystallization. This mechanism is consistent with the observed coarse, monomodal product and with the highest flux recorded for this seed size. The 75–125 µm case exhibits a lower surface area per mass, allowing supersaturation to persist for the longest, and promoting secondary nucleation through the collision and attrition of large seed crystals. For seeds with a diameter of 210–300 µm, the low surface area per mass allows supersaturation to persist the longest, promoting secondary nucleation through the collision and attrition of the large seed particles. This generates a fine mode superimposed on coarse seed growth, resulting in a bimodal CSD. Such bimodality is a classical symbol of seeded crystallization under conditions of attrition-enhanced secondary nucleation [31,32]. The trends observed here are consistent with fundamental principles of seeding. Which predict coarser and narrower CSDs for small, well-dispersed seeds, but broader or multimodal CSDs for larger, attrition-prone seeds [18,28]. This directly connects the CSD to process stability: the monomodal distribution achieved with the smallest seeds signifies supersaturation control, which not only produces coarser crystals but is also essential for protecting the membrane interface from scaling, thereby enabling the highest and most stable flux.

### 3.4. Hydrodynamic Regime Effects at Fixed Seeding

**Figure 8:**
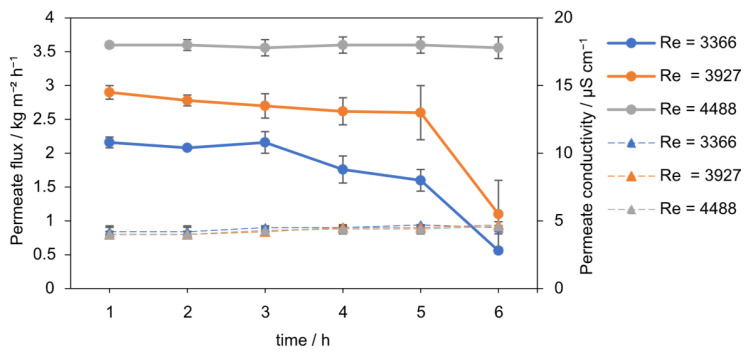
Permeate flux (solid line) over 6 h at different Reynolds numbers (*Re* = 3366, 3927, 4488, 0.1 g L^−1^ SiO_2_, 30–60 µm) with PTFE membrane. Lower *Re* led to flux decline from scaling, while *Re* = 4488 maintained stable performance. The permeate conductivity (dotted line) over time stays constant at around 4.5 µS cm^−1^.

**Figure 9:**
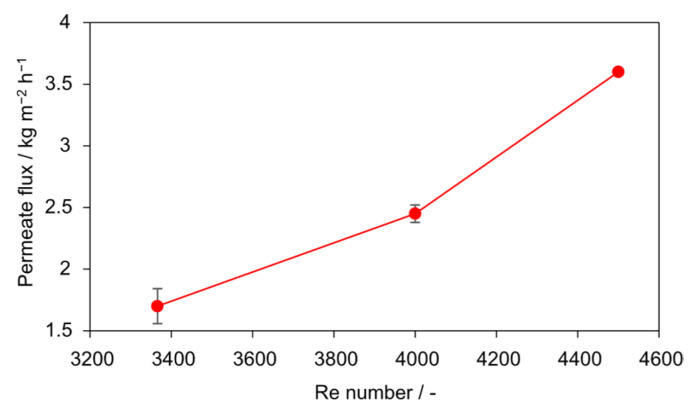
Influence of *Re* on steady-state permeate flux with PTFE membrane (0.1 g L^−1^ SiO_2_, 30–60 µm). Increasing *Re* from 3366 to 4488 resulted in a twofold flux enhancement.

A critical Reynolds number threshold for maintaining stable flux was identified, directly linking hydrodynamic conditions to process performance. The effect of *Re* number on permeate flux in seeded AGMDCr is presented in Figure 8 and Figure 9. At the lowest flow condition (*Re* = 3366), the flux declined continuously from 2.1 to 1 kg m^−2^ h^−1^ over 6 h (−52%). At an intermediate *Re* of 3927, the flux was initially higher, 2.8 kg m^−2^ h^−1^, but dropped sharply after 5 h, reaching 1.1 kg m^−2^ h^−1^ by the end of the run. In contrast, operation at *Re* = 4488 maintained a stable flux of 3.55 kg m^−2^ h^−1^ throughout, with no significant decline during the 6 h experiment.

The average fluxes over 6 h (Figure 9) emphasize the stabilizing role of turbulence. A clear increase is observed with *Re*: from 1.7 kg m^−2^ h^−1^ (*Re* = 3366) to 2.45 kg m^−2^ h^−1^ (*Re* = 3927) and 3.6 kg m^−2^ h^−1^ (*Re* = 4488). The improvement of more than 110% between the lowest and highest *Re* number highlights the critical importance of hydrodynamic shear. These findings indicate that for the present setup, a minimum threshold of *Re* = 4000–4500 is required to ensure stable flux and to suppress interfacial scaling. Because *Re* is a dimensionless, scale-independent parameter under geometric and fluid-property similarity, this finding provides a practical design criterion for industrial-scale AGMDCr modules. For scale-up, numbering-up (parallelization) is preferable to geometric scaling provided inlet/outlet hydraulics are comparable. Extrapolation beyond the tested range suggests that operating at Reynolds numbers well above 4500 drives the flux toward a plateau, yielding (diminishing returns) while pumping energy increases disproportionately. Accordingly, operation just above the turbulent threshold (*Re* ≈ 4500) balances stability and energy efficiency for this configuration.

At low *Re*, the hydrodynamic boundary layer is thick, and salt accumulates at the membrane surface, accelerating local supersaturation, scaling, and partial wetting. Increasing *Re* reduces the CP layer thickness and enhances shear at the membrane interface, thereby maintaining the vapor pressure driving force and is consistent with shifting crystallization into the bulk of the seeded suspension. Salt rejection remained high and stable under all tested conditions. Permeate conductivity was consistently around 4.5 µS cm^−1^ (R ≥ 99.99%), indicating that no macroscopic pore wetting occurred during any of the experiments. Thus, the observed flux declining at low and intermediate *Re* is primarily attributed to CP-induced scaling rather than wetting failure.

The findings align with earlier MD studies, which have shown that turbulence reduces boundary-layer thickness and delays interfacial crystallization [20,21]. However, combining turbulence with heterogeneous seeding represents a step forward. Taken together, the data suggest that the combination provides a complementary effect that outperforms either strategy alone. Seeding alone cannot prevent flux decline at low Re, as bulk nucleation is insufficient to offset strong CP near the membrane. Conversely, turbulence without seeding has been shown to mitigate polarization but not eliminate scaling. For this configuration, combining turbulence with heterogeneous seeding resulted in superior performance; at *Re* > 4500, flux was stable and salt rejection remained complete.

### 3.5. Post-Run Membrane Surface Morphology

**Figure 10:**
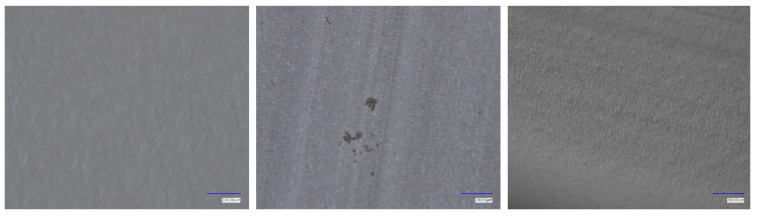
Surface images of PTFE membranes after AGMDCr operation: (**left**) pristine membrane, (**middle**) unseeded operation showing cubic NaCl deposits, and (**right**) seeded operation (0.1 g L^−1^ SiO_2_, 30–60 µm) showing a clean surface without scaling. The scale bar is 200 µm.

The final part of the study investigated PTFE membrane surface condition before use, after unseeded operation, and after seeded operation (0.1 g L^−1^, 30–60 µm) as shown in Figure 10. The pristine membrane (left) exhibits a homogenous fibrillated surface typical of expanded PTFE, with no visible deposits. After an unseeded operation (middle), distinct crystalline deposits appear, consistent with cubic NaCl morphology, confirming that supersaturation at the membrane surface led to heterogeneous nucleation and direct scaling on the hydrophobic interface. Such deposits explain the progressive flux and the sharp increase in permeate conductivity, as interfacial scaling reduces vapor transport and locally promotes pore wetting.

In contrast, after seeded operation (right), the membrane surface remains essentially free of visible salt deposits, retaining its pristine appearance. The absence of scaling confirms that the SiO_2_ seeds successfully shifted nucleation into the bulk, thereby reducing interfacial supersaturation and suppressing heterogeneous nucleation on the membrane. This observation corroborates the stable flux and low permeate conductivity observed during the seeded runs.

The microstructural evidence, therefore, provides direct support for the proposed mechanism: seeding particles reduce concentration polarization and act as preferential nucleation sites, thereby protecting the membrane surface from salt deposition. Similar trends have been reported in previous seeded MDCr studies, where shifting crystallization away from the membrane interface enhanced flux stability and minimized fouling [18].

## 4. Conclusions

This study systematically evaluated heterogeneous SiO_2_ seeding in AGMDCr with hypersaline NaCl brine (300 g L^−1^) using PP and PTFE membranes, establishing quantitative design guidelines for combining seeding and hydrodynamics. Compared to unseeded operation, an optimal seeding concentration of 0.1 g L^−1^ (30–60 µm) significantly improved performance for both membranes, increasing steady-state flux by 41% for PTFE (to 3.51 from 2.49 kg m^−2^ h^−1^) and by 17% for PP (to 1.92 from 1.63 kg m^−2^ h^−1^). This enhancement improved 6 h water recovery from 33% to 54% for PTFE, and from a premature run-end at 11% (4 h) to a stable 20% for PP, all while maintaining salt rejection ≥ 99.99%. Seed size and concentration influenced the crystal size distribution, with the smallest seeds (30–60 µm) producing the coarsest, monomodal products, which correlated with the highest measured flux. Hydrodynamic testing identified a critical threshold (*Re* ≥ 4000), where turbulent operation more than doubled the average flux compared to lower-Re runs and maintained stable performance. Post-run microscopy confirmed that seeded operations maintained a clean PTFE membrane surface, whereas unseeded runs resulted in cubic NaCl scaling deposits. While seeding was effective for both materials, PTFE consistently outperformed PP due to its larger pore structure and thinner wall. Overall, this work demonstrates that combining turbulent operation with optimized heterogeneous seeding enables effective control of scaling and wetting, advancing MDCr as a feasible ZLD technology for hypersaline brines.

## Figures and Tables

**Table 1:** The properties of the used PP and PTFE membranes.

Properties	Units	PTFE	PP
Inner diameter	mm	8	5.5
Outer diameter	mm	10	8.8
Wall thickness	mm	1	1.55
Pore size	mm	0.1–0.2	0.2
Length	m	1	1
Membrane area	m^2^	0.025	0.017
Porosity	%	70–80	73

## Data Availability

The data presented in this study are available on request from the corresponding author.

## Abbreviations

The following abbreviations are used in this manuscript:

AGMDCr	Air gap membrane distillation crystallization
CP	Concentration polarization
CSD	Crystal size distribution
MD	Membrane distillation
MDCr	Membrane distillation crystallization
PP	Polypropylene
PTFE	Polytetrafluoroethylene
Re	Reynolds number
TP	Temperature polarization
ZLD	Zero-liquid-discharge
